# Identification of Novel lncRNAs in Ovarian Cancer and Their Impact on Overall Survival

**DOI:** 10.3390/ijms22031079

**Published:** 2021-01-22

**Authors:** Nicholas Cardillo, Douglas Russo, Andreea Newtson, Henry Reyes, Yasmin Lyons, Eric Devor, David Bender, Michael J. Goodheart, Jesus Gonzalez-Bosquet

**Affiliations:** 1 Department of Obstetrics and Gynecology, University of Iowa Hospitals and Clinics, Iowa City, IA 52245, USA; douglas-russo@uiowa.edu (D.R.); andreea-newtson@uiowa.edu (A.N.); eric-devor@uiowa.edu (E.D.); david-bender@uiowa.edu (D.B.); michael-goodheart@uiowa.edu (M.J.G.); jesus-gonzalezbosquet@uiowa.edu (J.G.-B.); 2 Department of Gynecologic Oncology, University of Buffalo, Buffalo, NY 14203, USA; hdreyes@buffalo.edu; 3 Department of Gynecologic Oncology, University of Texas—San Antonio, San Antonio, TX 78229, USA; lyonsy@uthscsa.edu

**Keywords:** long non-coding RNA, ovarian cancer, overall survival, chemotherapy response

## Abstract

Long non-coding RNA’s (lncRNA) are RNA sequences that do not encode proteins and are greater than 200 nucleotides in length. They regulate complex cellular mechanisms and have been associated with prognosis in various types of cancer. We aimed to identify lncRNA sequences that are associated with high grade serous ovarian cancer (HGSC) and assess their impact on overall survival. RNA was extracted from 112 HGSC patients and 12 normal fallopian tube samples from our Biobank tissue repository. RNA was sequenced and the Ultrafast and Comprehensive lncRNA detection and quantification pipeline (UClncR) was used for the identification of lncRNA sequences. Univariate logistic and multivariate lasso regression analyses identified lncRNA that was associated with HGSC. Univariate and multivariate Cox proportional hazard ratios were used to evaluate independent predictors of survival. 1943 of 16,325 investigated lncRNA’s were differentially expressed in HGSC as compared to controls (*p* < 0.001). Nine of these demonstrated association with cancer after multivariate lasso regression. Our multivariate analysis of survival identified four lncRNA’s associated with survival in HGSC. Three out of these four were found to be independently significant after accounting for all clinical covariates. Lastly, seven lncRNAs were independently associated with initial response to chemotherapy; four portended a worse response, while three were associated with improved response. More research is needed, but there is potential for these lncRNAs to be used as biomarkers of HGSC or predictors of treatment outcome in the future.

## 1. Introduction

The majority of ovarian cancer patients are diagnosed in Stage III and IV, leading to a five-year overall survival of approximately 40% [1,2]. The current recommended treatment preferably includes a cytoreductive surgery, with the removal of all visible disease, followed by six cycles of combined chemotherapy with a platinum agent and paclitaxel. In patients with contraindications to cytoreductive surgery, a regimen consisting of neoadjuvant chemotherapy followed by cytoreductive surgery and at least three cycles of adjuvant chemotherapy, with or without the addition of bevacizumab, is warranted [3,4,5]. While approximately 75% of patients will respond to frontline chemotherapy, those that do not respond have a poor prognosis, and they will typically die from their disease within two years [6]. Currently, we are unable to predict which patients will respond to this initial regimen prior to administration, so we cannot identify those patients that may benefit more from alternative treatments. Therefore, there is a critical need to understand which biological processes are associated with response to chemotherapy. Subsequently, we could design alternative regimens for non-responder patients targeting those processes. The complicated transcription environment of ovarian cancer must be decoded in order to achieve this goal. Various mechanisms have been implicated in chemotherapy response, including drug efflux, tolerance to oxidative stress, alterations of DNA repair mechanisms, or resistance to apoptosis [7]. They can occur through both genetic and epigenetic regulation and may involve DNA methylation, as well as modifications in expression of both coding and non-coding RNA [8,9,10].

Non-coding RNA can be categorized into two groups: long and small. Small non-coding RNA includes microRNAs, small interfering RNA, and PIWI-interacting RNAs, and they are implicated in the initiation and progression of various diseases [11,12]. Long non-coding RNAs (lncRNA) are sequences of more than 200 nucleotides that have myriad functions in transcription and regulation [13]. They can upregulate or downregulate protein expression through their interactions with mRNA or microRNA, or they can affect protein function by directly binding with the protein itself [14]. This class of RNA represents a novel field of investigation into how cancers respond to chemotherapy. Many lncRNAs have been linked to drug response in various types of cancer, including breast, melanoma, urothelial, and ovarian [15]. A better understanding of the association of lncRNAs with response to chemotherapy in ovarian cancer could lead to novel therapeutic targets and prediction models of chemo-response.

The advent of high-throughput transcription technologies and software analytical pipelines has been of great benefit in identifying lncRNA in cancers. One analytical software tool, the Ultrafast and Comprehensive lncRNA detection pipeline (UClncR), has been validated as an accurate and fast method for evaluating large volumes of lncRNA [16]. This system streamlines the process of identifying and predicting lncRNAs within submitted RNA specimens and it provides a full array of lncRNAs within those specimens. This allows for a fast way to perform large scale genomic comparison. We hypothesize that specific lncRNA’s are involved in the regulation of response to chemotherapy, impacting survival in ovarian cancer patients. To test our hypothesis, we aimed to assess the differential lncRNA expression between patients with high-grade serous ovarian cancer (HGSC) and normal fallopian tube specimens. We then compared lncRNA expression in those HGSC that respond versus those that do not respond to initial chemotherapy. Subsequently, we evaluated the association of lncRNAs on survival.

## 2. Results

### 2.1. Association with HGSC

**Figure 1:**
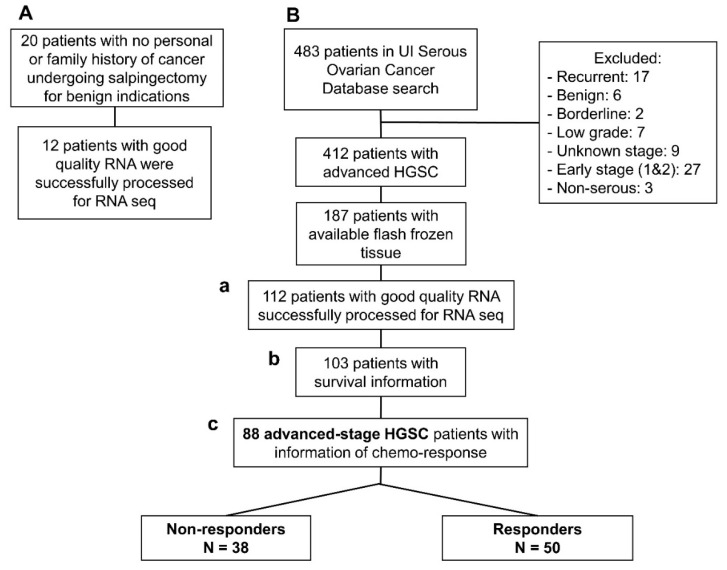
Flow-chart of patients included in this study: (**A**) Normal fallopian tube samples from women undergoing contraceptive procedures. (**B**) Patients with high-grade serous ovarian cancer (HGSC) from the Women’s Health Tissue Repository: (**a**) 112 patients with HGSC and successful RNA-seq that were included in the comparison with lncRNA extracted from normal tubes; (**b**) 103 HGSC patients with successful RNA-seq that had outcome and follow-up data necessary for the survival analysis; and, (**c**) 88 advanced stage, high-grade, HGSC with data about chemotherapy response based on the definition stablished in the Methods section.

**Figure 2:**
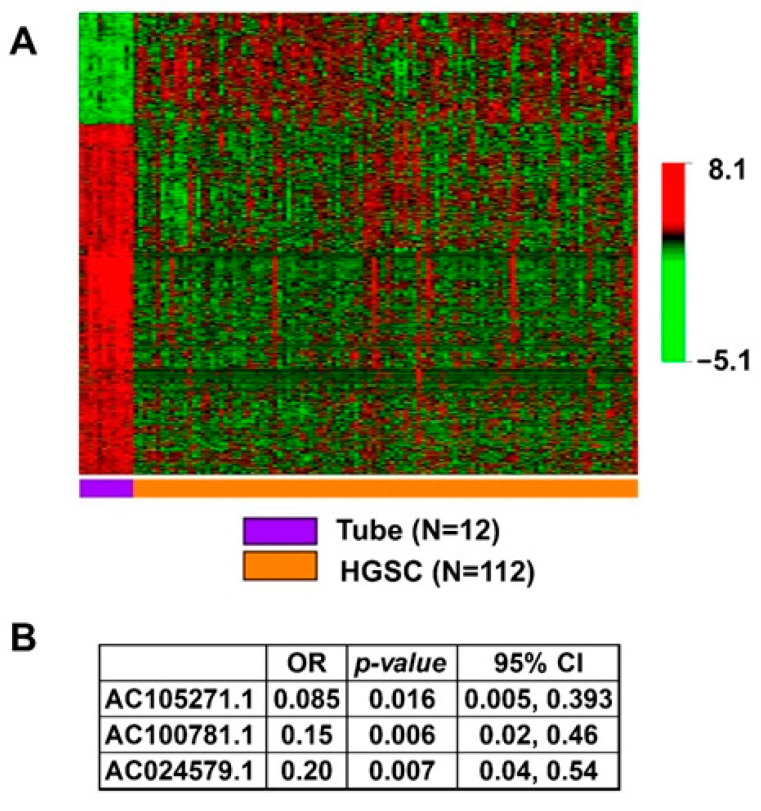
Association of lncRNA with HGSC. (**A**) Heatmap showing statistically significant, differential lncRNA expression between HGSC and normal tube in the univariate analysis with *p* < 0.001, *N* = 1943. (**B**) Independently significant lncRNA in the multivariable model of association with HGSC.

We identified 16,325 lncRNA sequences from the extracted RNA of 112 HGSC and 12 normal tube samples (Figure 1). In Figure 2A, we present the 1943 lncRNA sequences that were differentially expressed in HGSC as compared to normal Fallopian tube controls (*p* < 0.001). Of these 1943 sequences, three were significant after the multivariate logistic regression analysis (Figure 2B). All three demonstrated decreased expression; evidence of decreased regulation within the tumor.

### 2.2. Association of lncRNAs with Survival

**Figure 3:**
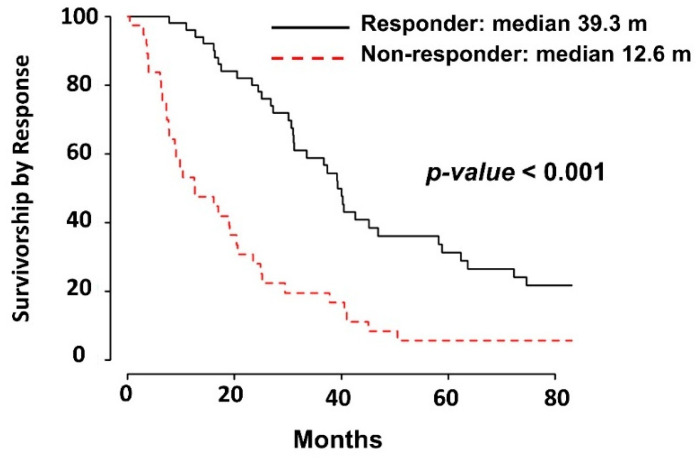
Kaplan–Meier survival curve based on response to chemotherapy: Median survival of 39.3 months for responders and 12.6 for non-responders.

Demographic, clinical, and outcome data, including survival and genomic data from RNA sequencing (RNA-seq), were available for 103 HGSC patients (Figure 1(Bb)). Univariate cox proportional hazard ratios revealed clinical factors that are associated with survival, including: age (HR: 1.03; *p* = 0.002) Charlson comorbidity index (HR 1.21; *p* = 0.004), residual disease after surgery (HR 2.31; *p* = 0.008), neoadjuvant chemotherapy (HR 2.92; *p* < 0.001), and response to chemotherapy (HR 4.55; *p* < 0.001) (to see more details about other variables see Table 1). These are all consistent with previously reported data. These significant clinical variables were introduced into the multivariate analysis of survival (Table 2). Only two clinical variables were independent in the Cox proportional Hazard ratio multivariate model: Charlson comorbidity index, and the response to chemotherapy (Figure 3).

**Figure 4:**
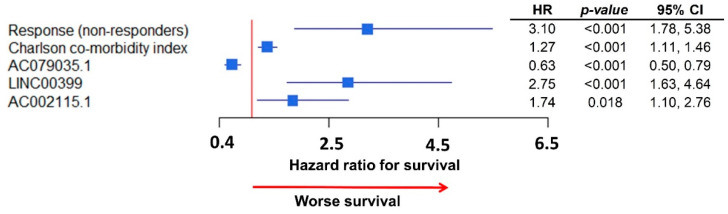
Forest plot of the integrative multivariate model with clinical data and lncRNA expressions: Response to chemotherapy remained highly associated (HR: 3.1) with survival; LINC00399 was the lncRNA with highest association with survival (HR: 2.75).

In the multivariate analysis of survival for lncRNA expression, we found four unique lncRNAs to be independently significant (Table 2). Subsequently, we integrated all independent clinical and lncRNA variables in a single multivariate model of survival (Figure 4). Response to chemotherapy, Charlson co-morbidity Index, as well as three lncRNAs, AC079035.1, LINC00399, and AC002115.1 were all associated with survival in HGSC patients, as represented in the forest plot shown in Figure 4. The strongest association with decreased survival were for response to chemotherapy, HR 3.1 (95% CI: 1.78, 5.38) and for LINC00399 expression, HR 2.75 (95% CI: 1.63, 2.76).

### 2.3. Association of lncRNAs with Chemo-Response

**Figure 5:**
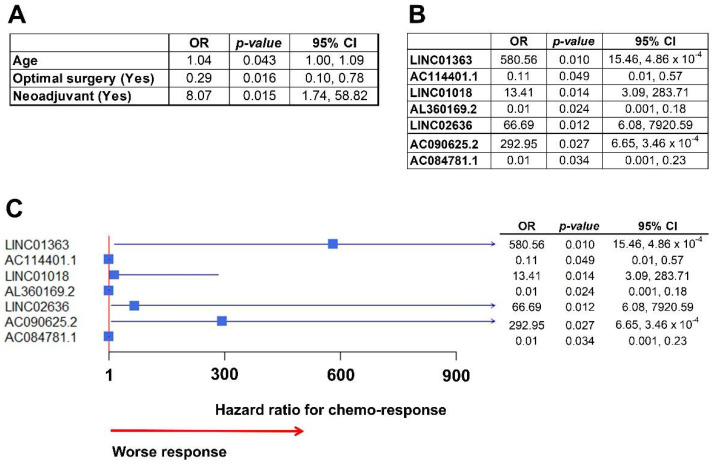
Association of lncRNA with chemo-response. (**A**) Table with clinical variables associated with chemo-response in the multivariate analysis. Patients that received neoadjuvant chemotherapy before surgery had an increased risk of not responding to initial chemotherapy (HR: 8.07), based on the definition described in Methods. (**B**) Table with lncRNA independently associated with chemo-response after the multivariate analysis. (**C**) Forest plot of the integrative multivariate model with clinical data and lncRNA expressions. All clinical variables dropped from the model and only lncRNAs remained. Notably, there were three lncRNAs highly associated with response to chemotherapy (worse response): LINC01363, with a HR over 580; LINC02636, with a HR over 66; and, ACO90625.2, with a HR over 292.

Table 3 presents the baseline clinical, pathological, and outcome data from HGSC patients based on their response to initial standard chemotherapy. Differences between both groups of patients (responders versus non-responders) for all of these variables were assessed. Subsequently, significant variables were introduced in a multivariate logistic model to assess differences between groups. Age, optimal surgery (with <1 cm residual disease after initial surgery), and administration of neoadjuvant chemotherapy before surgery were independently significant (Figure 5A).

In the multivariate analysis to assess the association between lncRNA expression and response to chemotherapy, seven lncRNAs were significantly associated with chemo-response (Figure 5B). Subsequently, we built an integrative multivariate logistic model, including independently significant clinical and molecular variables (lncRNAs). When put together only lncRNAs remained independently significant, and the clinical variables dropped from the model (represented in Figure 5C forest plot). Notably, three lncRNAs were highly associated with worse chemo-response, with OR over 65 (LINC02636) and up to 580 (LINC01363).

### 2.4. Validation of Analyses in the Cancer Genome Atlas (TCGA) HGSC Dataset

**Figure 6:**
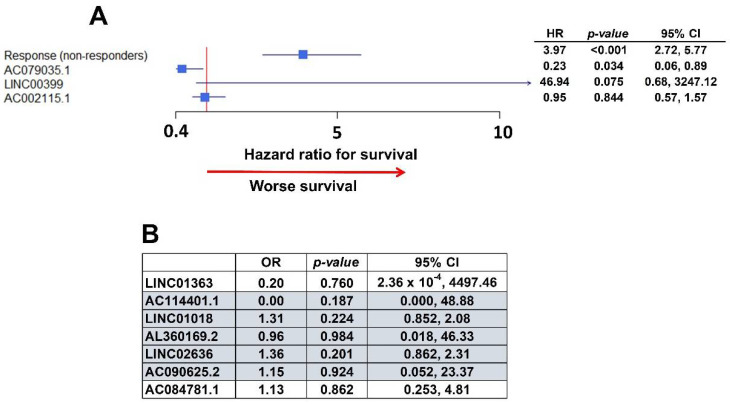
Validation within The Cancer Genome Atlas (TCGA) ovarian cancer dataset. (**A**) Forest plot with the multivariate analysis of survival in TCGA data. AC079035.1 and LINC00399 are associated with better and worse survival, respectively, as they were in the initial University of Iowa (UI) analysis. (**B**) Table with multivariate analysis of chemo-response in TCGA data. Highlighted lncRNAs had the same association with chemo-response as in the initial UI model: OR smaller than one is an association with worse chemo-response, greater than one represents better response to chemotherapy.

The survival association analysis was performed in the TCGA database. Unfortunately, not all of the covariates were available in the dataset, such as the Charlson co-morbidity index. In the multivariate analysis with the existing data (Figure 6A), non-responders to chemotherapy also had significantly worse survival, and AC079035.1 conferred significantly better survival, as observed in the initial analysis (Figure 3, Table 2). Additionally, LINC00399 presented a trend to better survival, although not statistically significant. AC002115.1 was not significant.

In the analysis to validate lncRNAs that are associated with chemo-response (Figure 5), five of the lncRNAs identified in our data were found to have the same type of response in the TCGA dataset (Figure 6B): LINC01018, LINC02636, and AC090625.2 were associated with worse response, while AC114401.1 and AL360169.2 were associated with a better response to initial chemotherapy. However, none of them achieve statistical significance (Figure 6B).

## 3. Discussion

We analyzed the entire lncRNA profile of our HGSC and normal fallopian tube specimens and found three lncRNAs to be significantly associated with HGSC in the multivariate regression model. These lncRNAs have not previously been described as being associated with HGSC. All of these showed decreased expression, which indicates that these lncRNAs may be involved with regulation or tumor suppression and their decreased expression facilitates rampant dysregulation and subsequent tumorigenesis.

Additionally, we combined our lncRNA dataset with clinically relevant data in order to assess the association of lncRNA expression with survival. There were three lncRNAs independently associated with survival after the multivariate analysis and after accounting for all other significant clinical variables. Of these three, two were associated with worse overall survival and one was associated with improved survival. AC002115.1, which was associated with better survival, is present in a region encoding for HAUS5, which is related to microtubule organization and is essential for mitosis [17,18]. It is unknown whether AC002115.1 directly interacts with HAUS5, but their proximity on the genome is notable. The other significant lncRNAs identified have unknown functions and are uninvestigated. Interestingly, our analysis did not identify lncRNAs that had previously been reported to be associated with ovarian cancer, such as UCA1, HOTAIR, XIST, or H1913 [15].

In the integrated multivariate analysis of association with chemo-response, we identified four sequences independently associated with worse response and three sequences that are associated with improved response, even after accounting for significant clinical co-variates. LINC01363, which is related to worse prognosis, has been shown to be upregulated in breast, ovarian, and cervical cancer [19]. However, most interestingly, LINC01018, which was associated with a worse response to initial chemotherapy in our model, has been linked to progression in hepatocellular carcinoma (HCC) [20]. LINC01018 is downregulated in HCC, thereby decreasing the expression of FOXO1, which prevents apoptosis. The overexpression of LINC01018 leads to decreased proliferation and increased apoptosis in hepatocellular carcinoma and it is associated with overall survival [21]. This same association has been demonstrated in non-small cell lung carcinoma [22]. It is likely that LINC01018 serves a similar function in ovarian cancer. None of the other lncRNA’s identified by this study have a known function or association. This demonstrates the great limitations in our current knowledge regarding the function and importance of lncRNA.

In our study, we have identified distinct groups of lncRNA that are associated with HGSC, with survival and with response to chemotherapy. LncRNAs have been involved in different functions concerning cancer [13], including, as we had observed, genesis, survival, and response to treatment. A potential explanation is that different lncRNA regulate different mechanisms that can affect cancer at different stages. For example, those that were found to be different between tubal and HGSC could be associated with mechanisms that are involved early in the process of cancer genesis. Other groups affecting survival may affect intrinsic mechanisms that are used for the host to fight invasion. A specific group of these lncRNA may be those associated with therapy response. This shows the complexity of interactions that regulate neoformation and cancer progression. In our study, we just touched the surface, and further studies are needed in order to assess the regulatory function of these lncRNA in cancer formation, progression, and response to treatment and how these mechanisms interact. Deeper knowledge of these regulatory mechanisms may evolve into better and more diverse targeted treatments to these processes.

Our analysis did not find previously identified lncRNAs, such as UCA1, HOTAIR, XIST, and H1913, to be associated with survival or chemo-response. In previous reports, HOTAIR was associated with survival in patients that were previously treated with carboplatin, but this difference disappears in patients that are naïve to the drug [23]. All of our patients were platinum-naïve at the time the samples were collected. Additionally, we assessed associations with survival and chemo-response for thousands of lncRNAs and we reported those that were independently associated with outcomes. The stronger effect of some lncRNAs may have overcome other weaker associations in the multivariate analysis. Similarly, UCA1 is significantly up-regulated with taxol-resistant ovarian cancer [24]. The expression of UCA1 is increased in patients who develop taxol resistance after initial chemotherapy treatment. We would not expect to see the upregulation of UCA1 in chemo-naïve patients, as our study only used samples from ovarian cancer patients at initial diagnosis.

One of the strengths of our study is that we were able to evaluate all of the lncRNAs expressed in our specimens while using high throughput transcription. Additionally, our controls were fallopian tube samples of benign histology, from women with no family history of breast or ovarian cancer. We were also able to use thorough clinical data in order to control for significant variables impacting survival and/or chemo-response. Consequently, our study is free from the confirmation bias of analyzing a single lncRNA, which has been identified in other cancers and then assessing its expression in ovarian cancer. Finally, we were able to validate some of these results in an independent HGSC dataset, TCGA. Despite some limitations of TCGA clinical information, we found that some of the lncRNAs that are associated with survival and response to initial chemotherapy in the initial UI analysis, were also significant in TCGA multivariate models. To our knowledge, we are the first to present such a comprehensive analysis of lncRNA expression combined with clinical data to assess for associate, survival, and chemotherapy response in ovarian cancer.

Our study may be limited by a relatively small sample size of controls. However, the strong conservation of lncRNA expression within normal controls likely means that more controls would not add much to our analysis. The paucity of research in lncRNA biological functions, specifically for those that are described in our study, limit the conclusions that we can draw regarding these associations with survival and chemotherapy response.

lncRNA are important regulators of the genome and they serve a significant function in tumorigenesis in ovarian cancer. Isolating those lncRNAs that are specific to ovarian cancer and affect survival and response to chemotherapy will hopefully lead to molecular profiles for individual patients that guide providers toward which treatment regimens will be most effective and improve patient outcomes. While lncRNA functions are rapidly being elucidated, there are many thousands of them, and their interactions are complex. Nevertheless, with further research, there are exciting opportunities for improved ovarian cancer outcomes within this field.

## 4. Materials and Methods

This is a retrospective case-control study that aims to assess the role of lncRNA in HGSC outcomes and response to treatment. In order to determine the response to chemotherapy, we classified HGSC patients as responders or non-responders. Responders were those with a progression-free survival of at least six months after the first platinum-based treatment. Non-responders were those who had evidence of disease within six months of their platinum-based treatment (platinum-resistant) or experienced disease progression during treatment (platinum-refractory).

### 4.1. Tissue Procurement and Processing

Tissue samples and clinical outcome data were obtained from the Department of Obstetrics and Gynecology Gynecologic Oncology Bank (IRB, ID#200209010), which is part of the Women’s Health Tissue Repository (WHTR, IRB, ID#201804817). All of the tissues archived in the Gynecologic Oncology Bank were originally obtained from adult patients under informed consent in accordance with University of Iowa IRB guidelines. 112 HGSC specimens provided adequate RNA for analysis.

We additionally collected 12 fallopian tube samples from women undergoing contraceptive procedures. Fallopian tubes were obtained from patients with no family history of cancer beside squamous cell carcinoma of the skin and who were undergoing salpingectomy for benign indications (mainly sterilization). DNA and RNA were extracted from epithelial tissue coming from the junction of the ampullary and fimbriated end of fallopian tubes. Twenty normal fallopian tube specimens were obtained. Of those, 12 produced viable RNA for analysis. RNA from both the fallopian tube and HGSC specimens had already been extracted and purified in a previous study [25].

### 4.2. Clinical Data

Clinical and pathological data were collected from the electronic medical record. Clinical variables that were previously observed to be associated with chemo-response were included in the data collection [26]. Only baseline clinical and pathological characteristics that can be obtained before starting initial chemotherapy were included.

### 4.3. RNA Sequencing

Of the 187 patients identified in the original HGSC panel, 112 tumor tissues with sufficient RNA yield and quality were available for analysis (Figure 1(Ba)). The total cellular RNA was purified from primary tumor tissue using the mirVana (Thermo Fisher, Waltham, MA, USA) RNA purification kit following the manufacturers’ instructions. The yield and quality of purified cellular RNA was assessed using a Trinean DropSense 16 spectrophotometer and an Agilent Model 2100 bioanalyzer. Samples with an RNA integrity number (RIN) greater than or equal to 7.0 were selected for RNA sequencing [27].

The equal mass total RNA (500 ng) from each qualifying tumor was fragmented, converted to cDNA, and then ligated to bar-coded sequencing adaptors while using Illumina TriSeq stranded total RNA library preparation (Illumina, San Diego, CA, USA). Molar concentrations of the indexed libraries were confirmed on the Agilent Model 2100 bioanalyzer and libraries were then combined into equimolar pools for sequencing. The concentration of the pools was confirmed while using the Illumina Library Quantification Kit (KAPA Biosystems, Wilmington, MA, USA). Sequencing was then carried out on the Illumina HiSeq 4000 genome sequencing platform using 150 bp paired-end SBS chemistry. All of the library preparation and sequencing were performed in the Genome Facility of the University of Iowa Institute of Human Genetics (IIHG).

### 4.4. lncRNA Detection

The Ultrafast and Comprehensive lncRNA detection and quantification pipeline (UClncR) was used for the identification of lncRNA expression in both HGSC tissue samples and control fallopian tubes [16]. Briefly, sequence reads in fastq format were aligned to the human reference genome (hg38) while using HISAT2, which resulted in aligned and sorted BAM files [28]. Aligned BAM files were then processed by UClncR with intact GENCODE annotation (v19) to recover the known lncRNAs. UClncR performs transcript assembly, predicts lncRNA candidates, quantifies, and annotates both known and novel lncRNA candidates, and generates a convenient report for downstream analysis. The UClncR main results are lncRNA expression matrix tables, which include known lncRNAs at raw and normalized values. Matrices with normalized expression values were used for posterior statistical analyses.

### 4.5. Statistical Analysis

#### 4.5.1. Association with HGSC

A table with all lncRNAs and their annotation was constructed for all HGSC and tubal samples. Student’s *t*-test was used in order to compare lncRNA expression in HGSC samples to those of normal fallopian tube controls. Those lncRNA that were identified as significant (*p* < 0.001, to account for multiple comparisons) in the univariate analysis were introduced in the multivariate model. A multivariable regression model was used in order to assess differences for these lncRNA found to be significant in the univariate analysis.

#### 4.5.2. Association with Survival

Survival analysis of lncRNAs was performed using Cox proportional hazard ratios (HR). A multivariate analysis of survival was built by introducing significant variables in the univariate analysis (*p* < 0.05) in a Cox Proportional HR multivariate model. The survival assessments of clinical variables were also performed using Cox proportional HR. Subsequently, we created a multivariate Cox model with significant (*p* < 0.05) clinical characteristics. Independently significant lncRNAs and clinical variables from their respective multivariate Cox models were both introduced in an integrative multivariate Cox model of survival.

#### 4.5.3. Association with Chemo-Response

Differences between clinical variables in responders and non-responders were assessed with logistic regression. *p*-values < 0.05 were considered to be statistically significant. Association analysis of lncRNAs with chemo-response (previously defined) was performed while using lasso multivariate regression and including all 16,325 lncRNA expressions. In order to account for all clinical variables associated with chemo-response, we performed a multivariate logistic regression of all significant variables in the univariate analysis (*p* < 0.05). Afterwards, independently significant clinical variables were introduced in an integrative regression model with those lncRNAs resulting from the lasso regression.

#### 4.5.4. Validation of Analysis within TCGA Ovarian Cancer Dataset

Validation of lncRNA associations with survival and chemo-response was performed while using the TCGA serous ovarian cancer dataset. BAM files were downloaded from the TCGA website. These files were the product of aligning sequences to the human reference genome (hg38). Aligned BAM files were then processed by UClncR with intact GENCODE annotation (v19), as described before. Subsequently, lncRNA expression matrix tables with normalized values were used for statistical analyses. Only those values that were found to be significant in the UI analyses were validated in TCGA.

## Figures and Tables

**Table 1:** Patient characteristics and association with survival: Out of the 122 patients with HGSC, 103 had complete clinical and outcomes information for the survival analysis.

			HGSC Patients	*p*-Value
			*N* = 103	
Age	(Mean)		59.8	0.002 *
Charlson Comorbidity Index	1–3		18	0.004 *
	4–6		64	
	>6		16	
FIGO Stage	2		3	0.995
	3		68	
	4		25	
Disease in Upper abdomen (Other than Omentum) by Imaging	Yes	Large Bowel (*N* = 4)	63	0.089
		Porta—Hepatis (*N* = 5)		
		Mesenteric Mets (*N* = 4)		
		Other (*N* = 26)		
	No		40	
Disease in the Chest by Imaging	Yes	Chest (*N* = 5)	7	0.936
		Pleural effusion (*N* = 5)		
	No		96	
Grade	2		21	0.555
	3		67	
Residual disease after surgery	Microscopic		20	0.008 *
	Macroscopic		82	
	Optimal (<1 cm)		64	0.105
	Suboptimal (>1 cm)		36	
Removal of Pelvic LN	Yes		17	0.089
	No		86	
Removal of Para-Aortic LN	Yes		10	0.144
	No		93	
Surgical complexity score	Low		52	0.789
	Intermediate		47	
	High		4	
Neoadjuvant Chemotherapy	Yes		13	<0.001 *
	No		88	
Number of Cycles delivered	<6		1	0.194
	≥6		42	
Dose Dense Chemotherapy	Yes		3	0.354
Response to Chemotherapy	Yes		50	<0.001 *
	No		38	

* Statistically significant (later included in the multivariate analysis).

**Table 2:** Multivariate analysis of lncRNA with survival: The top of the table has clinical variables associated independently with survival in the multivariate analysis: only two of all variables significant in the univariable analysis of survival (variables in Table 1). Non-responders to initial chemotherapy have more than four-fold risk of dying from the disease. With each point increase of the Charlson co-morbidity Index, the risk of dying from disease is 19% higher. On the lower part of the table are those lncRNA associated independently with survival after the multivariate analysis.

	HR	*p*-Value	95% CI
**Multivariate model with clinical data**			
Charlson Index	1.19	0.009	1.04, 1.36
Response (non-responders)	4.17	<0.001	2.50, 6.96
**Multivariate model with lncRNA**			
AC079035.1	0.63	<0.001	0.50, 0.79
LINC00399	2.42	<0.001	1.49, 3.94
AL139021.1	1.49	0.020	1.06, 2.08
AC002115.1	2.91	<0.001	1.89, 4.49

**Table 3:** Patient characteristics and association with chemo-response: Patients were divided between responders and non-responders based on definitions described in the methods. We had a total of 88 advanced-stage HGSC patients with clinical information regarding response to chemotherapy and a minimum follow-up of six months after treatment that were used for this analysis (Figure 1(Bc)).

			Responders	Non-Responders	*p*-Value
			*N* = 50	*N* = 38	
Age			56	64	0.009 *
Charlson Comorbidity Index	1–3		9	4	0.068 *
	4–6		35	21	
	>6		1	6	
FIGO Stage	3		39	25	0.069
	4		7	12	
Disease in Upper abdomen (Other than Omentum) by Imaging	Yes	Large Bowel (*N* = 4)	28	29	0.051
		Porta—Hepatis (*N* = 4)			
		Mesenteric Mets (*N* = 3)			
		Other (*N* = 22)			
	No		22	9	
Disease in the Chest by Imaging	Yes	Chest (*N* = 4)	6	0	0.992
		Pleural effusion (*N* = 5)			
	No		44	38	
Grade	2		8	11	0.875
	3		35	23	
Residual disease after surgery	Microscopic		12	3	0.053
	Macroscopic		37	35	
	Optimal (<1 cm)		37	20	0.039 *
	Suboptimal (>1 cm)		13	18	
Removal of Pelvic LN	Yes		9	4	0.333
	No		41	34	
Removal of Para-Aortic LN	Yes		5	3	0.734
	No		45	35	
Surgical complexity score	Low		22	23	0.990
	Intermediate		28	12	
	High		0	3	
Neoadjuvant Chemotherapy	Yes		2	10	0.009 *
	No		47	28	
Number of Cycles delivered	<6		2	8	0.344
	≥6		48	30	
Dose Dense Chemotherapy	Yes		1	1	0.844

* Statistically significant (later included in the multivariate analysis).

## Data Availability

Datasets with RNA-seq have been submitted to the GEO at NCBI website: https://www.ncbi.nlm.nih.gov/geo/, and can be browsed by their accession number: GSE156699. Software utilized by this study is also publicly available at Bioconductor website: http://bioconductor.org/.
